# Supplementary material for: Accurately programming complex light regimes with multichannel LEDs
Source: Quant Plant Biol. 2026 Mar 5;7:e6. doi: 10.1017/qpb.2026.10041 (PMC13078106; doi:10.1017/qpb.2026.10041)
Supplement: Vong et al. supplementary material [file S2632882826100411sup001.zip › Supplemental_Figures (2).pdf]

# Supplemental Figures

## S1

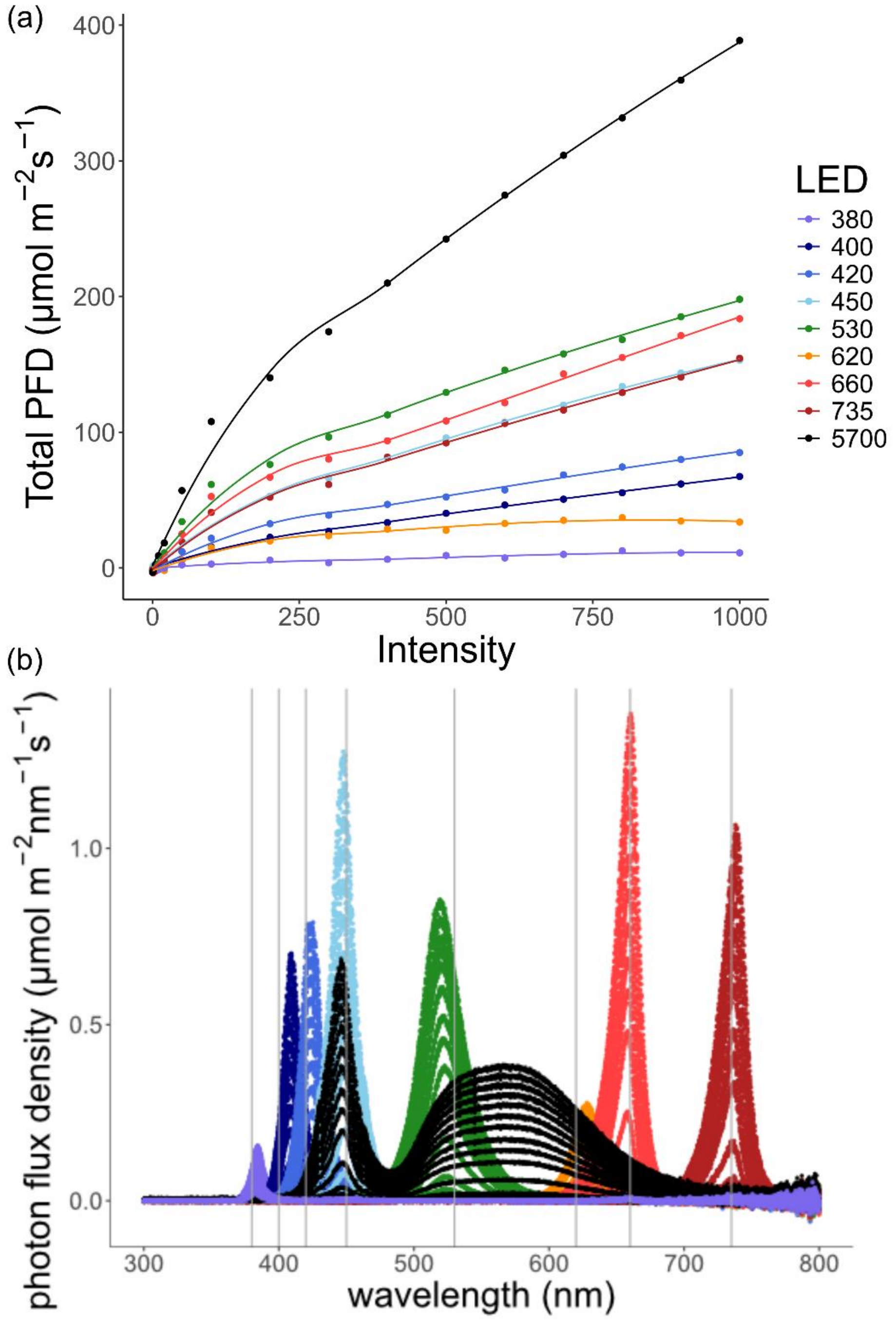


**Figure S1.** Calibrating the lights. **(a)** Spectrum produced by LEDs as each channel was calibrated at increasing intensities. The vertical lines represent the purported wavelengths. **(b)** Total irradiance (across the spectrum) at each intensity.

# S2

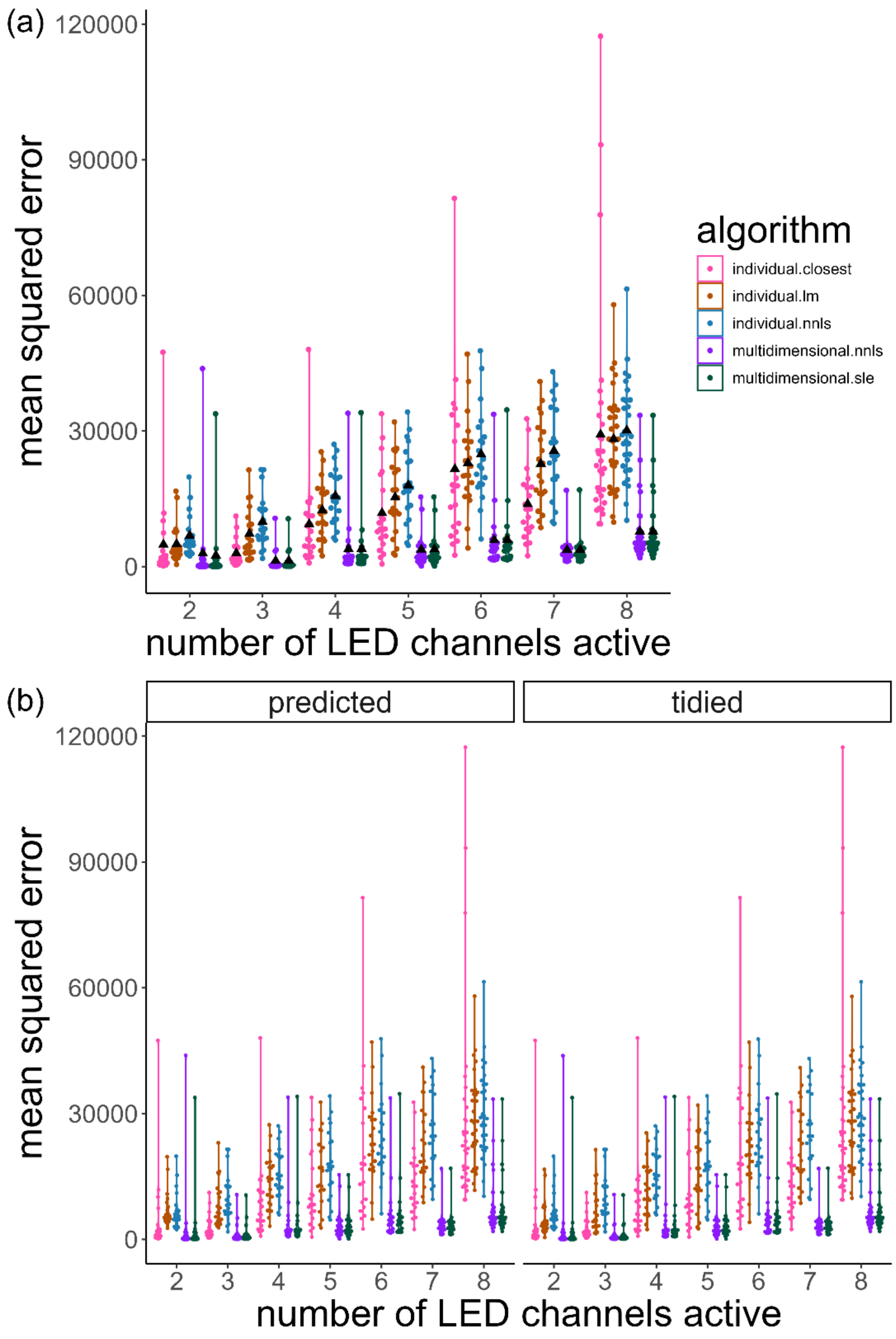


**Figure S2.** Algorithm comparisons. ▲ denotes the mean. **(a)** Mean squared error of indivIdual and multidimensional algorithms at different complexity levels. **(b)** Mean squared error of the raw predictions of the various algorithms vs after the final tidying step.

S3

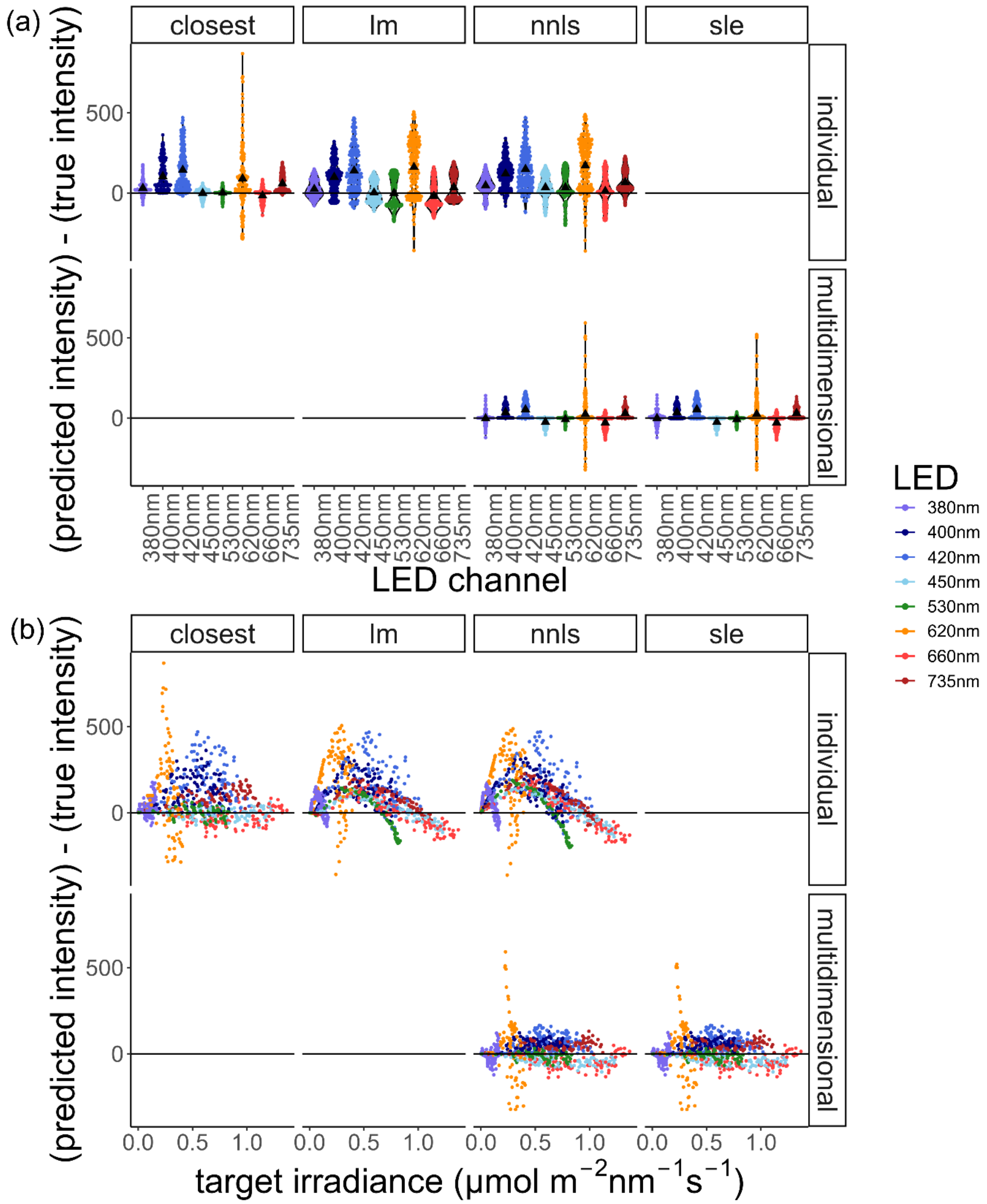
(a)
closest
lm
nnls
sle
individual
multidimensional
500
0
(predicted intensity) - (true intensity)
380nm
400nm
420nm
450nm
530nm
620nm
660nm
735nm
LED channel
(b)
0.0 0.5 1.0
target irradiance (μmol m−2nm−1s−1)
LED
380nm
400nm
420nm
450nm
530nm
620nm
660nm
735nm

**Figure S3.** Comparing the performance of the algorithms at the LED level. **(a)** The distribution of residuals for each of the algorithms. ▲denotes mean. **(b)** The relationship between residuals and target irradiance.

## S4

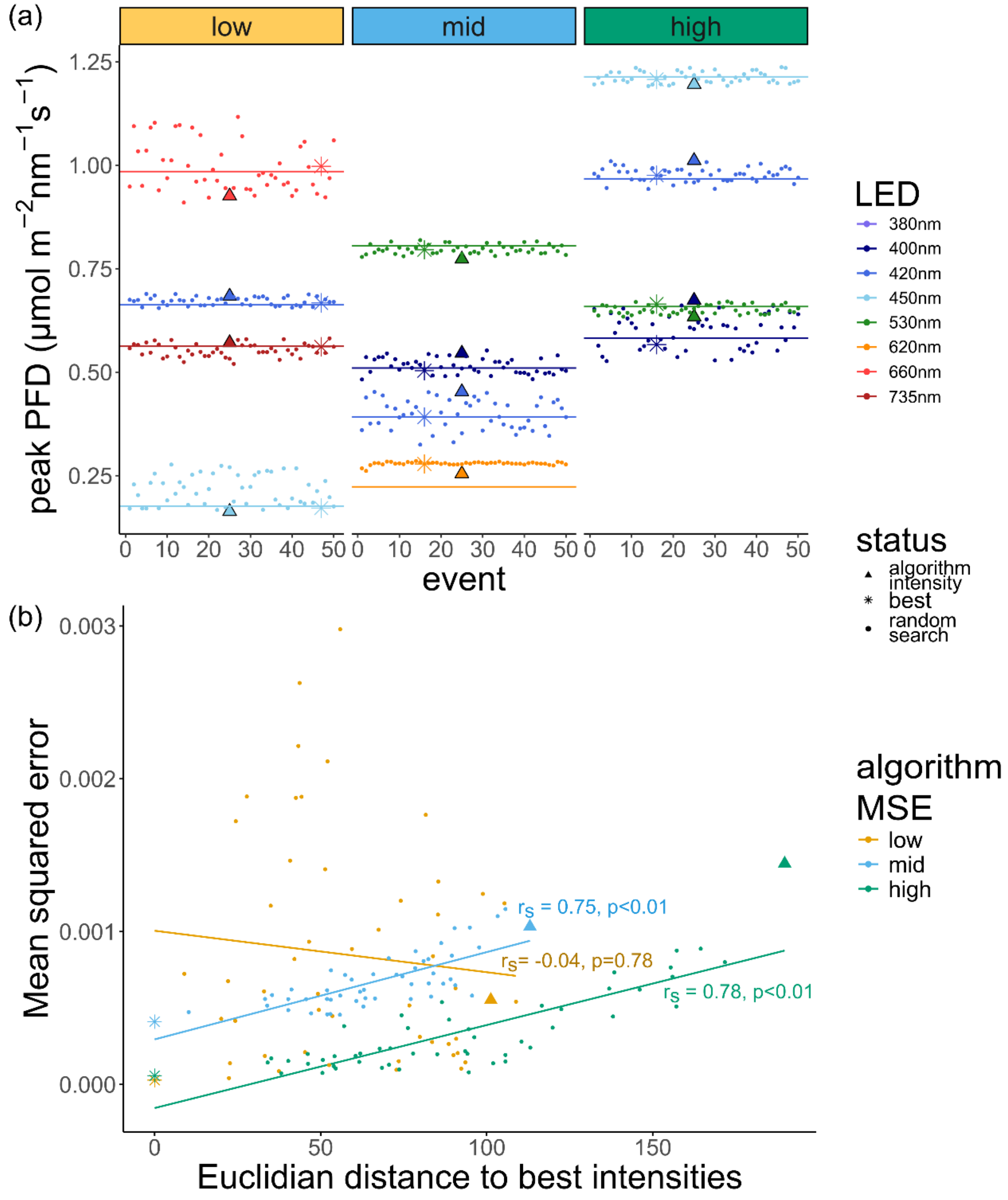


**Figure S4.** Random search. ▲ is the starting point (i.e. intensities predicted by the multidimensional NNLS), * is the best intensity (i.e. lowest mean squared error) found through random search. **(a)** The progression of the random search for each of the starting MSEs (low, medium high). The horizontal line (-) marks the target irradiance for

each of the LEDs. **(b)** The relationship between the straight line distance (i.e. euclidian distance) to best intensities (across all LEDs involved), and the mean squared error. Each point represents an event, with a linear regression (solid line, -) and Spearman rank correlation coefficient (rs) for each starting MSE.

S5

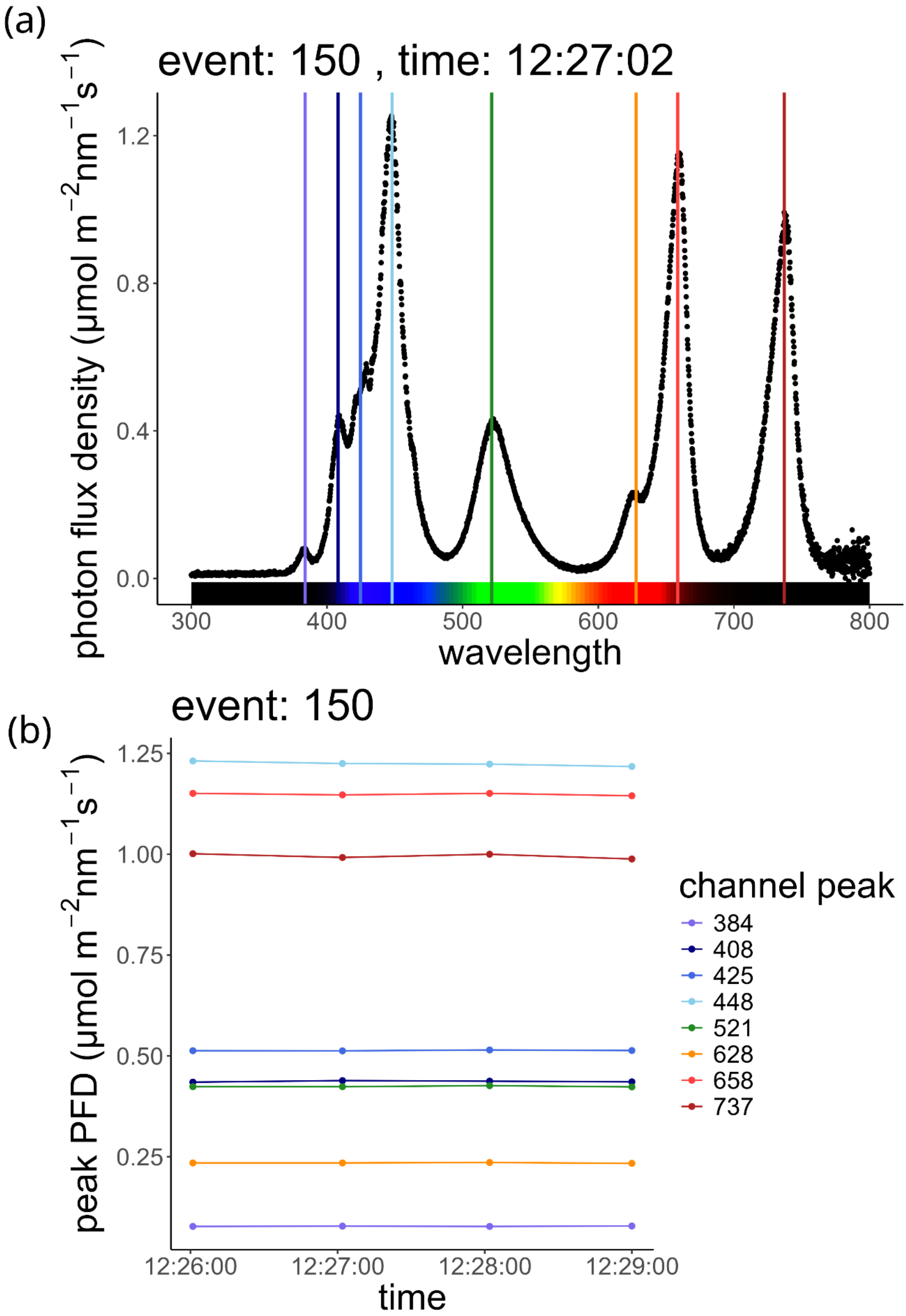
(a)
event: 150 , time: 12:27:02
photon flux density (μmol m−2nm−1s−1)
1.2
0.8
0.4
0.0
300
400
500
600
700
800
wavelength
(b)
event: 150
peak PFD (μmol m−2nm−1s−1)
1.25
1.00
0.75
0.50
0.25
12:26:00
12:27:00
12:28:00
12:29:00
time
channel peak
384
408
425
448
521
628
658
737

**Figure S5.** Spectral data from event 150  (last column) of Supplementary Table S2. Measurements of the LED channels at random intensities were used as targets for testing the algorithm. **A)** Spectrum when 8 LED channels were on simultaneously, with vertical lines representing the peak wavelength of a given LED channel. **B)** PFD of the peak wavelengths during event 150, with the same intensity settings throughout.
