# Supplementary material for: Accurately programming complex light regimes with multichannel LEDs
Source: Quant Plant Biol. 2026 Mar 5;7:e6. doi: 10.1017/qpb.2026.10041 (PMC13078106; doi:10.1017/qpb.2026.10041)
Supplement: Vong et al. supplementary material [file S2632882826100411sup001.zip › LightFitR_GetStarted_vignette (2).pdf]

LightFitR

Introduction

LightFitR is an R package for designing complex light regimes with LED lights. Often, these light fixtures are programmed with ‘intensity’ units, which often does not scale linearly with the actual measured light output from the light fixtures. Further, if using multiple wavelength channels, there will often be bleedthrough between the channels, affecting the quality and quantity of light received by your experimental subjects. Our package aims to combat both of these challenges. It takes calibration data and user-defined target irradiances and it tells you what intensities to use in order to achieve those irradiances.

Note that this package does not support broad spectrum white LEDs

Terminology

This package uses ‘intensity’ to mean the unitless settings that the light fixture uses.

‘irradiance’ means the measured light output from the fixture.This can be in the users’ preferred light units, provided it is used consistently throughout.

‘regime’ refers to a repeating daily schedule that the light fixtures run on.

‘event’ refers to the point in the light regime when the lights change intensity.

Getting started

You will need several inputs:

- calibration data from your light fixture
- target irradiances that you want your lights to achieve.
- timepoints for your events

Calibration data

Your calibration data must have these 4 columns:

- LED channel: which channel the given measurement corresponds to
- Intensity: the intensity setting used on that channel to achieve the measurement
- Wavelength: the wavelength of the measurement (if using a light meter which doesn’t measure irradiance across multiple wavelengths, set the wavelength to the peak wavelength of the channel)
- Irradiance: The measured light output on that LED channel at that intensity. This can be in any units your measurement device uses, provided you use the same units throughout.

For more information about how to collect calibration data, see https://doi.org/10.1101/2025.06.06.658293

Here is an example of what the calibration data could look like:

```
calibration <- LightFitR::calibration
head(calibration)
#>                                                           filename     time
#> 7780 Apollo_Calib_20240827__AbsoluteIrradiance__103__00-52-02-433.txt 00:52:02
#> 7781 Apollo_Calib_20240827__AbsoluteIrradiance__103__00-52-02-433.txt 00:52:02
#> 7782 Apollo_Calib_20240827__AbsoluteIrradiance__103__00-52-02-433.txt 00:52:02
```

```
#> 7783 Apollo_Calib_20240827__AbsoluteIrradiance__103__00-52-02-433.txt 00:52:02
#> 7784 Apollo_Calib_20240827__AbsoluteIrradiance__103__00-52-02-433.txt 00:52:02
#> 7785 Apollo_Calib_20240827__AbsoluteIrradiance__103__00-52-02-433.txt 00:52:02
#>      led intensity wavelength irradiance
#> 7780 380       500    300.001       0.01
#> 7781 380       500    300.213       0.02
#> 7782 380       500    300.426      -0.01
#> 7783 380       500    300.639      -0.03
#> 7784 380       500    300.852      -0.01
#> 7785 380       500    301.064       0.02
```

Target irradiance

The target irradiances are a set of irradiances that you wish to achieve for your experiment.

This should be a matrix, with rows representing LED channels and columns representing timepoints or events.

For example:

```
target <- LightFitR::target_irradiance
print(target)
#>       [,1] [,2] [,3] [,4] [,5] [,6] [,7] [,8] [,9] [,10]
#> 380nm  2.8  3.6  2.6  0.4  1.8  3.4  2.0  0.8  3.8   2.6
#> 400nm  4.8 17.8 18.0 13.6 18.0 11.2 18.2  1.6 18.4  19.6
#> 420nm 14.2  5.0  1.2  8.2  1.6 16.4  7.0 15.4 16.0   8.4
#> 450nm 20.4 23.2 15.0 28.4  6.2 21.6  1.2 27.2 33.6  14.6
#> 530nm  4.4  5.2 11.8 10.4  1.2 10.4  5.2 19.0  7.4  17.6
#> 620nm  0.2  0.8  1.4  2.2  2.4  3.4  0.0  4.8  4.8   1.0
#> 660nm  4.0 15.6  8.0  9.2 17.8 11.8 18.8  3.0 23.0  18.6
#> 735nm  1.0 14.2 17.0 17.0  7.6  6.0 16.0  9.8  6.6   0.6
#> 5700k  0.0  0.0  0.0  0.0  0.0  0.0  0.0  0.0  0.0   0.0
```

Event timepoints

This should be a vector with a length the same as the column number of your target irradiances.

Each event timepoint should be in the POSIXct format. The date can be arbitrary as the package only cares about the timestamp. We recommend the `lubridate` package for working with times.

For example:

```
times <- LightFitR::time_vector
print(times)
#>  [1] "1970-01-01 00:00:00 GMT" "1970-01-01 00:05:00 GMT"
#>  [3] "1970-01-01 00:10:00 GMT" "1970-01-01 00:15:00 GMT"
#>  [5] "1970-01-01 00:20:00 GMT" "1970-01-01 00:25:00 GMT"
#>  [7] "1970-01-01 00:30:00 GMT" "1970-01-01 00:35:00 GMT"
#>  [9] "1970-01-01 00:40:00 GMT" "1970-01-01 00:45:00 GMT"
```

Creating the regime of intensities

Now you have all the inputs, let's make a regime:

```
regime <- makeRegime(times, target, calibration$led, calibration$wavelength,
         calibration$intensity, calibration$irradiance)
#> Ranges fall within irradiances acheivable by heliospectra: TRUE
#> Warning in internal.closestWavelength(unique(calibration_df$wavelength), : We
#> couldn't find exact matches with the peak wavelengths specified. Returning the
#> closest wavelengths
#> Warning in internal.closestWavelength(unique(calib$wavelength), peaks): We
#> couldn't find exact matches with the peak wavelengths specified. Returning the
#> closest wavelengths
#> Ranges fall within irradiances acheivable by heliospectra: TRUE
```

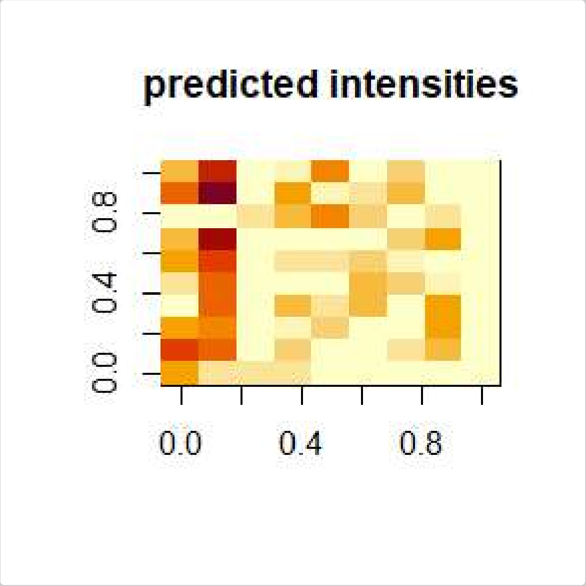


```
print(regime)
#>        00:00:00   00:05:00   00:10:00   00:15:00   00:20:00   00:25:00
#> time   "00:00:00" "00:05:00" "00:10:00" "00:15:00" "00:20:00" "00:25:00"
#> hour   "0"        "0"        "0"        "0"        "0"        "0"
#> minute "0"        "5"        "10"       "15"       "20"       "25"
#> second "0"        "0"        "0"        "0"        "0"        "0"
#> 380nm  "963"      "1000"     "1000"     "74"       "474"      "1000"
#> 400nm  "517"      "1000"     "1000"     "1000"     "1000"     "1000"
#> 420nm  "385"      "0"        "0"        "0"        "0"        "160"
#> 450nm  "493"      "633"      "278"      "856"      "51"       "549"
#> 530nm  "62"       "86"       "671"      "485"      "22"       "484"
#> 620nm  "6"        "49"       "43"       "777"      "821"      "600"
#> 660nm  "48"       "499"      "98"       "176"      "601"      "292"
#> 735nm  "12"       "838"      "1000"     "1000"     "278"      "166"
#> 5700k  "0"        "0"        "0"        "0"        "0"        "0"
#>        00:30:00   00:35:00   00:40:00   00:45:00
#> time   "00:30:00" "00:35:00" "00:40:00" "00:45:00"
#> hour   "0"        "0"        "0"        "0"
#> minute "30"       "35"       "40"       "45"
#> second "0"        "0"        "0"        "0"
#> 380nm  "817"      "50"       "1000"     "934"
#> 400nm  "1000"     "54"       "1000"     "1000"
#> 420nm  "0"        "525"      "0"        "0"
#> 450nm  "0"        "779"      "1000"     "261"
#> 530nm  "61"       "1000"     "201"      "1000"
```

```
#> 620nm  "0"      "629"    "527"    "9"
#> 660nm  "710"    "34"     "886"    "627"
#> 735nm  "1000"   "390"    "177"    "0"
#> 5700k  "0"      "0"      "0"      "0"
```

As you can see, the `makeRegime` function automatically creates a heatmap of your regime to allow the user to sanity check the intensity output.

It also creates a matrix of intensities for you to set your lights to. The layout is very similar to that of the target matrix. But with extra rows for the timepoints, since some models of lights requires that.

Exporting the regime

Currently, we can only export regimes compatible with Heliospectra DYNA(TM) lights running the R3.2.2-Release firmware.

```
write.helioSchedule(regime, filename='my_regime.txt', format='json')
```

If you do not use this model of light, you can still write the regime matrix to a csv. Or you're very welcome to write your own function to format the regime to be compatible with your model of lights!

How it works

`makeRegime` is the core user-facing function in this package. But you probably want to know what is happening behind the scenes. We will summarise below, and full explanation is available at: https://doi.org/10.1101/2025.06.06.658293

When you run `makeRegime`, it carries out 4 steps in the background:

1. Calculate closest intensities
2. Predict the intensities to use to achieve the target irradiance (via a system of linear equations or non-negative least squares)
3. Tidy the intensities (rounding to integer, keep within the range of intensities that the lights can be set to)
4. Format the intensities and timestamps into a human-readable regime matrix

We'll go through each of the steps below.

Closest intensities

This step searches through the calibration data to find intensities which produce the closest irradiances to your target irradiance.

Using the example target irradiances from above:

```
closest <- LightFitR::internal.closestIntensities(target, calibration[, c(3,5,4,6)])
#> Warning in internal.closestWavelength(unique(calibration_df$wavelength), : We
#> couldn't find exact matches with the peak wavelengths specified. Returning the
#> closest wavelengths
rownames(closest) <- LightFitR::helio.dyna.leds$name
print(closest)
#>       [,1] [,2] [,3] [,4] [,5] [,6] [,7] [,8] [,9] [,10]
#> 380nm 1000 1000  800   50  400 1000  400  100 1000   800
#> 400nm  600 1000 1000 1000 1000 1000 1000  100 1000  1000
```

```
#> 420nm  600  100   20  200   20  700  200  600  700   200
#> 450nm  500  700  300  900   50  600   10  800 1000   300
#> 530nm   50  100  700  500   20  500  100 1000  200  1000
#> 620nm   10   20   50 1000 1000  700    0  500  500    50
#> 660nm   50  500  100  200  600  300  600   50  900   600
#> 735nm   20  900 1000 1000  300  200 1000  400  200    10
#> 5700k    0    0    0    0    0    0    0    0    0     0
```

We've got a matrix of intensities for each channel and event!

To convince ourselves that these are indeed the closest, we can compare our target irradiances with the calibration data. Let's do this for the first event:

```
# Define variables

## Calibration
calib_wavelengths <- unique(calibration$wavelength)
calib_intensities <- unique(calibration$intensity)

## Subset the closest matrix to the first event
closest_first <- closest[,1]
print(closest_first)
#> 380nm 400nm 420nm 450nm 530nm 620nm 660nm 735nm 5700k
#>  1000   600   600   500    50    10    50    20     0

## Subset the targets
target_first <- target[,1]
print(target_first)
#> 380nm 400nm 420nm 450nm 530nm 620nm 660nm 735nm 5700k
#>   2.8   4.8  14.2  20.4   4.4   0.2   4.0   1.0   0.0

# Go through each channel of the first event
sanity_check <- sapply(1:length(closest_first), function(i){

  ## Set relevant variables
  tar <- target_first[i]
  clo <- closest_first[i]
  led <- helio.dyna.leds[i, 'wavelength']

  ## Subset calibration data to the LED at the peak wavelengths
  criteria <- calibration$led==led &
         calibration$wavelength==LightFitR:::internal.closestWavelength(calib_wavelengths, led)
  calib_subset <- calibration[criteria, 3:6]

  # Print outputs for user
  print(names(clo))
  print('This is the calibration data')
  print(calib_subset)
  print(paste('The target irradiance is', tar))
  print(paste('The closest intensity is', clo))
  print('---')

  return()
})
```

```
#> Warning in LightFitR:::internal.closestWavelength(calib_wavelengths, led): We
#> couldn't find exact matches with the peak wavelengths specified. Returning the
#> closest wavelengths
#> [1] "380nm"
#> [1] "This is the calibration data"
#>         led intensity wavelength irradiance
#> 8161    380       500    379.936       2.14
#> 21126   380       600    379.936       2.29
#> 34091   380       700    379.936       2.48
#> 49649   380       800    379.936       2.59
#> 60021   380       900    379.936       2.73
#> 72986   380      1000    379.936       2.79
#> 1120558 380         0    379.936      -0.02
#> 1291696 380         1    379.936      -0.03
#> 1405788 380         5    379.936       0.03
#> 1548403 380        10    379.936       0.08
#> 1691018 380        20    379.936       0.14
#> 1812889 380        50    379.936       0.48
#> 1823261 380       100    379.936       0.93
#> 1836226 380       200    379.936       1.37
#> 1849191 380       300    379.936       1.65
#> 1864749 380       400    379.936       1.90
#> [1] "The target irradiance is 2.8"
#> [1] "The closest intensity is 1000"
#> [1] "---"
#> Warning in LightFitR:::internal.closestWavelength(calib_wavelengths, led): We
#> couldn't find exact matches with the peak wavelengths specified. Returning the
#> closest wavelengths
#> [1] "400nm"
#> [1] "This is the calibration data"
#>        led intensity wavelength irradiance
#> 86048  400         0    399.932      -0.03
#> 99013  400         1    399.932      -0.02
#> 111978 400         5    399.932       0.03
#> 124943 400        10    399.932       0.14
#> 137908 400        20    399.932       0.31
#> 150873 400        50    399.932       0.86
#> 163838 400       100    399.932       1.78
#> 176803 400       200    399.932       2.40
#> 189768 400       300    399.932       3.07
#> 202733 400       400    399.932       3.61
#> 215698 400       500    399.932       4.26
#> 228663 400       600    399.932       4.81
#> 241628 400       700    399.932       5.35
#> 254593 400       800    399.932       5.79
#> 267558 400       900    399.932       6.12
#> 280523 400      1000    399.932       6.58
#> [1] "The target irradiance is 4.8"
#> [1] "The closest intensity is 600"
#> [1] "---"
#> Warning in LightFitR:::internal.closestWavelength(calib_wavelengths, led): We
#> couldn't find exact matches with the peak wavelengths specified. Returning the
#> closest wavelengths
#> [1] "420nm"
#> [1] "This is the calibration data"
```

```
#>        led intensity wavelength irradiance
#> 293586 420         0    419.989      -0.09
#> 306551 420         1    419.989      -0.05
#> 319516 420         5    419.989       0.18
#> 332481 420        10    419.989       0.46
#> 345446 420        20    419.989       1.09
#> 358411 420        50    419.989       3.09
#> 371376 420       100    419.989       5.83
#> 384341 420       200    419.989       7.75
#> 397306 420       300    419.989       9.55
#> 410271 420       400    419.989      11.30
#> 423236 420       500    419.989      13.09
#> 436201 420       600    419.989      14.59
#> 449166 420       700    419.989      16.24
#> 462131 420       800    419.989      17.63
#> 475096 420       900    419.989      18.99
#> 488061 420      1000    419.989      20.31
#> [1] "The target irradiance is 14.2"
#> [1] "The closest intensity is 600"
#> [1] "---"
#> [1] "450nm"
#> [1] "This is the calibration data"
#>        led intensity wavelength irradiance
#> 501174 450         0        450      -0.01
#> 516732 450         1        450       0.12
#> 527104 450         5        450       0.47
#> 540069 450        10        450       0.87
#> 553034 450        20        450       1.96
#> 568592 450        50        450       5.01
#> 578964 450       100        450       9.50
#> 591929 450       200        450      12.04
#> 604894 450       300        450      14.74
#> 617859 450       400        450      17.24
#> 630824 450       500        450      19.67
#> 643789 450       600        450      22.06
#> 656754 450       700        450      24.20
#> 669719 450       800        450      26.39
#> 682684 450       900        450      28.55
#> 695649 450      1000        450      30.61
#> [1] "The target irradiance is 20.4"
#> [1] "The closest intensity is 500"
#> [1] "---"
#> Warning in LightFitR:::internal.closestWavelength(calib_wavelengths, led): We
#> couldn't find exact matches with the peak wavelengths specified. Returning the
#> closest wavelengths
#> [1] "530nm"
#> [1] "This is the calibration data"
#>        led intensity wavelength irradiance
#> 709017 530         0    530.021       0.02
#> 721982 530         1    530.021       0.02
#> 734947 530         5    530.021       0.26
#> 747912 530        10    530.021       0.59
#> 760877 530        20    530.021       1.27
#> 773842 530        50    530.021       3.44
#> 786807 530       100    530.021       5.94
```

```
#> 799772 530       200    530.021       7.10
#> 812737 530       300    530.021       8.42
#> 825702 530       400    530.021       9.45
#> 838667 530       500    530.021      10.53
#> 851632 530       600    530.021      11.38
#> 864597 530       700    530.021      12.19
#> 877562 530       800    530.021      12.89
#> 890527 530       900    530.021      13.69
#> 903492 530      1000    530.021      14.43
#> [1] "The target irradiance is 4.4"
#> [1] "The closest intensity is 50"
#> [1] "---"
#> Warning in LightFitR:::internal.closestWavelength(calib_wavelengths, led): We
#> couldn't find exact matches with the peak wavelengths specified. Returning the
#> closest wavelengths
#> [1] "620nm"
#> [1] "This is the calibration data"
#>         led intensity wavelength irradiance
#> 916926  620         0    620.021      -0.06
#> 929891  620         1    620.021       0.02
#> 942856  620         5    620.021       0.07
#> 955821  620        10    620.021       0.21
#> 968786  620        20    620.021       0.47
#> 981751  620        50    620.021       1.38
#> 994716  620       100    620.021       2.61
#> 1007681 620       200    620.021       3.04
#> 1020646 620       300    620.021       3.48
#> 1033611 620       400    620.021       3.60
#> 1046576 620       500    620.021       3.69
#> 1059541 620       600    620.021       3.67
#> 1075099 620       700    620.021       3.46
#> 1088064 620       800    620.021       3.20
#> 1103622 620       900    620.021       2.90
#> 1116587 620      1000    620.021       2.51
#> [1] "The target irradiance is 0.2"
#> [1] "The closest intensity is 10"
#> [1] "---"
#> Warning in LightFitR:::internal.closestWavelength(calib_wavelengths, led): We
#> couldn't find exact matches with the peak wavelengths specified. Returning the
#> closest wavelengths
#> [1] "660nm"
#> [1] "This is the calibration data"
#>         led intensity wavelength irradiance
#> 1132359 660         0     659.97      -0.02
#> 1145324 660         1     659.97       0.01
#> 1160882 660         5     659.97       0.27
#> 1173847 660        10     659.97       0.69
#> 1189405 660        20     659.97       1.46
#> 1202370 660        50     659.97       4.07
#> 1217928 660       100     659.97       8.05
#> 1230893 660       200     659.97      10.15
#> 1246451 660       300     659.97      11.97
#> 1259416 660       400     659.97      13.70
#> 1274974 660       500     659.97      15.58
#> 1287939 660       600     659.97      17.59
```

```
#> 1303497 660       700     659.97      20.06
#> 1316462 660       800     659.97      21.67
#> 1334613 660       900     659.97      23.29
#> 1344985 660      1000     659.97      24.87
#> [1] "The target irradiance is 4"
#> [1] "The closest intensity is 50"
#> [1] "---"
#> Warning in LightFitR:::internal.closestWavelength(calib_wavelengths, led): We
#> couldn't find exact matches with the peak wavelengths specified. Returning the
#> closest wavelengths
#> [1] "735nm"
#> [1] "This is the calibration data"
#>         led intensity wavelength irradiance
#> 1360956 735         0    735.086      -0.02
#> 1373921 735         1    735.086       0.02
#> 1392072 735         5    735.086       0.14
#> 1402444 735        10    735.086       0.50
#> 1418002 735        20    735.086       1.01
#> 1430967 735        50    735.086       2.62
#> 1449118 735       100    735.086       5.26
#> 1459490 735       200    735.086       6.55
#> 1475048 735       300    735.086       7.66
#> 1488013 735       400    735.086       9.39
#> 1506164 735       500    735.086      10.64
#> 1516536 735       600    735.086      11.74
#> 1532094 735       700    735.086      12.62
#> 1545059 735       800    735.086      13.78
#> 1563210 735       900    735.086      14.45
#> 1573582 735      1000    735.086      15.40
#> [1] "The target irradiance is 1"
#> [1] "The closest intensity is 20"
#> [1] "---"
#> Warning in LightFitR:::internal.closestWavelength(calib_wavelengths, led): We
#> couldn't find exact matches with the peak wavelengths specified. Returning the
#> closest wavelengths
#> [1] "5700k"
#> [1] "This is the calibration data"
#>          led intensity wavelength irradiance
#> 1589509 5700         0    799.994      -0.08
#> 1602474 5700         1    799.994       0.01
#> 1618032 5700         5    799.994       0.07
#> 1630997 5700        10    799.994      -0.01
#> 1646555 5700        20    799.994      -0.11
#> 1659520 5700        50    799.994       0.03
#> 1675078 5700       100    799.994      -0.06
#> 1688043 5700       200    799.994       0.21
#> 1703601 5700       300    799.994       0.31
#> 1716566 5700       400    799.994       0.48
#> 1732124 5700       500    799.994       0.47
#> 1745089 5700       600    799.994       0.46
#> 1760647 5700       700    799.994       0.32
#> 1773612 5700       800    799.994       0.68
#> 1789170 5700       900    799.994       0.39
#> 1802135 5700      1000    799.994       0.58
#> [1] "The target irradiance is 0"
```

```
#> [1] "The closest intensity is 0"
#> [1] "---"
rm(sanity_check)
```

These closest intensities are used in the next step.

Predict intensities using SLE or NNLS

This step predicts the intensities to use, with a system of linear equations or non-negative least squares (a fancy version of SLE).

First, it uses the closest intensities and calibration data to create a matrix of irradiances for each event. Think of this as a heatmap with all of the bleedthrough between all the channels of the LED at the closest intensity. Again, we'll show this for the first event:

```
# Define variables
peakWavelengths <- LightFitR:::internal.closestWavelength(unique(calibration$wavelength),
        helio.dyna.leds[-9, 'wavelength'])
#> Warning in
#> LightFitR:::internal.closestWavelength(unique(calibration$wavelength), : We
#> couldn't find exact matches with the peak wavelengths specified. Returning the
#> closest wavelengths
firstEvent <- data.frame(led=LightFitR::helio.dyna.leds[-9, 'wavelength'],
        closest=closest_first[-9], intended=target_first[-9])
rm(closest_first, target_first)

# closest matrix

mat <- sapply(1:nrow(firstEvent), function(j){
  criteria <- (calibration$led == firstEvent[j, 'led']) & (calibration$intensity == firstEvent[j,
        'closest']) & (calibration$wavelength %in% peakWavelengths) # We want the irradiances
        (from calibration data) of each LED at the intensity where it is closest to the intended
        irradiance
  calibration[criteria, 'irradiance']
})

print(mat)
#>       [,1]  [,2]  [,3]  [,4]  [,5]  [,6]  [,7]  [,8]
#> [1,]  2.79  0.11  0.01  0.08 -0.03 -0.03 -0.01  0.00
#> [2,]  0.41  4.81  0.35  0.08  0.01 -0.03 -0.03 -0.01
#> [3,]  0.02  4.66 14.59  1.04 -0.08 -0.08 -0.02 -0.08
#> [4,]  0.11  0.29  0.95 19.67  0.05 -0.06  0.00  0.03
#> [5,]  0.02  0.04  0.04  0.07  3.44  0.01 -0.01  0.00
#> [6,] -0.04 -0.01 -0.04  0.01  0.01  0.21  0.14 -0.03
#> [7,]  0.08  0.00 -0.01  0.02  0.00 -0.03  4.07  0.01
#> [8,]  0.11  0.07 -0.02  0.18 -0.02  0.06  0.06  1.01
image(mat)
```

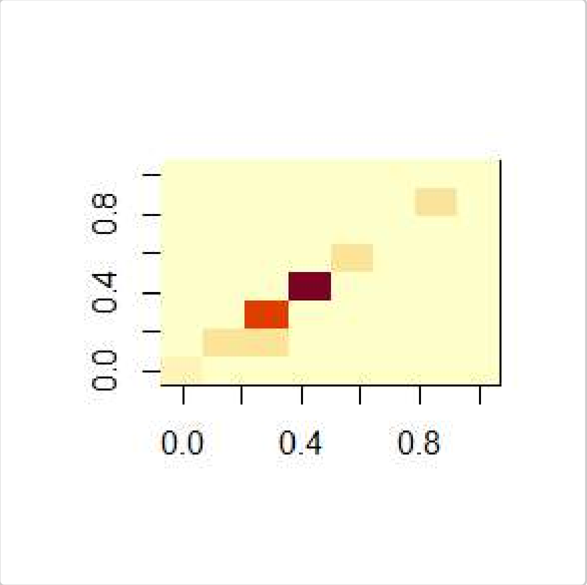

The leading diagonal is quite intense (which is what we want!). But as you can see, there is some bleedthrough between the channels, indicated by colour outside the leading diagonal.

Next, we make a model either using NNLS or SLE (more on the differences below). It solves the equation Ax=b, where A is the closest irradiance matrix (above), b are the target irradiances and x are unknown coefficients.

For the first event, it looks like this:

```
mod <- nnls::nnls(mat, firstEvent[,'intended'])
print(mod)
#> Nonnegative least squares model
#> x estimates: 0.9626349 0.8614317 0.6413636 0.985943 1.236892 0.6350962 0.9638369 0.5920488
#> residual sum-of-squares: 0
#> reason terminated: The solution has been computed sucessfully.
```

Finally, we use the coefficients (x, solved by the model) as well as the closest intensities to calculate the intensities we need to set the lights to:

```
intensities <- mod$x * firstEvent[, 'closest']
print(intensities)
#> [1] 962.634923 516.859030 384.818139 492.971513  61.844577   6.350962  48.191847
#> [8]  11.840976
```

Great! We have our intensities! We can put that straight into the lights right?

Well, most lights only accept whole numbers. And, although not apparent in this example, sometimes we predict intensities which are impossible for the lights (e.g. above 1000 in the case of Heliospectra DYNAs). So this brings us onto the next step!

Tidying

This is exactly as it sounds. We round our predicted intensities to the nearest integer, cap the predicted intensities to the maximum the lights can achieve (inferred from the calibration data), set any negative predictions to 0.

```
tidied <- LightFitR:::internal.tidyIntensities(intensities, calib_intensities)
print(tidied)
```

```
#> [1] 963 517 385 493  62   6  48  12
```

Much better!

Formatting

This final step just takes the resultant matrix and makes it more human readable. It adds row and column names, as well as the event timepoints. You can't really show it with just the first event, and you already saw the tidied matrix in the 'get started' example. So there isn't really any code to show for this step…

System of linear equations (SLE) vs Non-negative least squares (NNLS)

From our testing, there isn't much of a difference between SLE and NNLS when it comes to predicted intensities (https://doi.org/10.1101/2025.06.06.658293), except for occasional outliers. So if you're not happy with the intensities predicted by one method, try the other. The default for the package is NNLS but this was an arbitrary decision on our part.

SLE

This is the 'simpler' of the two methods. It solves the equation Ax=b straightforwardly. However, for us, this means it can predict intensities below 0 (i.e. unachievable by the lights since 0 means the light is off) and intensities above the maximum (again, impossible on the lights).

NNLS

This uses the Lawson-Hanson method to solve Ax=b in such a way that the predicted intensities are non-negative. This gets around the below 0 predictions, but can still predict intensities above the maximum. See the `nnls` package for more info on how NNLS works.
